# Systematic approach to contextualize findings of flexible endoscopic evaluation of swallowing in neurogenic dysphagia– towards an integrated FEES report

**DOI:** 10.1186/s42466-024-00321-8

**Published:** 2024-05-09

**Authors:** Rainer Dziewas, Tobias Warnecke, Bendix Labeit, Inga Claus, Paul Muhle, Stephan Oelenberg, Sigrid Ahring, Christina Wüller, Anne Jung, Jonas von Itter, Sonja Suntrup-Krueger

**Affiliations:** 1 Department of Neurology and Neurorehabilitation, Klinikum Osnabrück– Academic Teaching Hospital of the University of Münster, Am Finkenhügel 1, 49076 Osnabrück, Germany; 2 Department of Neurology, Medical Faculty, University Hospital Düsseldorf, Düsseldorf, Germany; 3 Department of Neurology with Institute for Translational Neurology, University Hospital Münster, Münster, Germany

**Keywords:** FEES, FEES report, Dysphagia, Neurogenic dysphagia

## Abstract

Flexible endoscopic evaluation of swallowing (FEES) is one of the most important methods for instrumental swallowing evaluation. The most challenging part of the examination consists in the interpretation of the various observations encountered during endoscopy and in the deduction of clinical consequences. This review proposes the framework for an integrated FEES-report that systematically moves from salient findings of FEES to more advanced domains such as dysphagia severity, phenotypes of swallowing impairment and pathomechanisms. Validated scales and scores are used to enhance the diagnostic yield. In the concluding part of the report, FEES-findings are put into the perspective of the clinical context. The potential etiology of dysphagia and conceivable differential diagnoses are considered, further diagnostic steps are proposed, treatment options are evaluated, and a timeframe for re-assessment is suggested. This framework is designed to be adaptable and open to continuous evolution. Additional items, such as novel FEES protocols, pathophysiological observations, advancements in disease-related knowledge, and new treatment options, can be easily incorporated. Moreover, there is potential for customizing this approach to report on FEES in structural dysphagia.

## Introduction

Flexible Endoscopic Evaluation of Swallowing (FEES) was first described in 1988 by the American speech and language pathologist (SLP) Susan Langmore and defined as a procedure separate from conventional otorhinolaryngoscopy, which lacks evaluation of swallowing [1]. Since then, FEES has been taken up by clinicians all around the world. Thus, FEES is regularly performed by SLPs and different medical professions, in particular neurologists, phoniatricians, otolaryngologists, geriatricians, pediatricians and intensivists, and is used in a variety of settings, such as outpatient care, acute-care hospitals, including stroke units and intensive care units, rehabilitation facilities and nursing homes [2–4]. Over the last years, education in FEES has undergone systematic formalization in various countries, including the United States [5], Great Britain [6], Germany [3, 7] and Japan [8]. Notably, the European Society for Swallowing Disorders (ESSD) has developed a transnational and multidisciplinary education program [9]. Therefore, along with the Videofluoroscopic Swallowing Study (VFSS), FEES today is the most commonly adopted method for instrumental dysphagia evaluation [2]. In terms of day-to-day practicality, FEES offers several advantages: First, it can be conducted at the bedside, allowing examination of severely motor-impaired, bedridden, or uncooperative patients—common scenarios in settings like the intensive care unit or stroke unit. Second, follow-up examinations can be promptly and, if necessary, frequently performed. Third, the assessment of oropharyngeal secretion management and the efficacy of cleaning mechanisms, such as coughing and throat clearing, can be conducted simply and directly. Lastly, therapeutic maneuvers can be implemented and assessed with immediate visual feedback for the care-giver and patient.

**Fig. 1:**
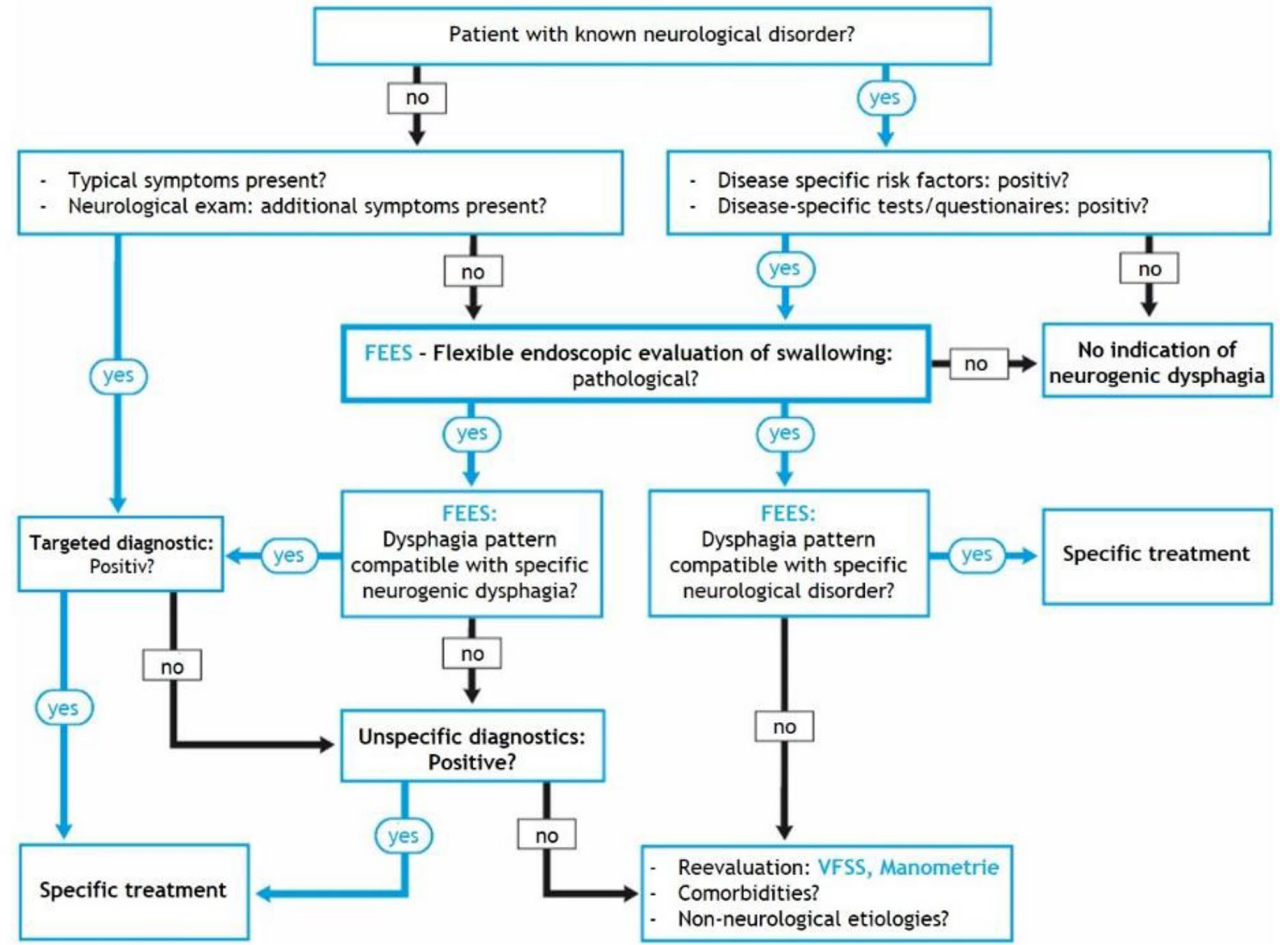
Structured algorithm for the diagnosis of neurogenic dysphagia [10] (FEES = Flexible Endoscopic Evaluation of Swallowing; VFSS = Videofluoroscopic Swallowing Study)

Due to these benefits, FEES holds a pivotal position in the diagnostic algorithm for neurogenic dysphagia, which offers a structured approach to effectively manage this challenging condition (Fig. 1) [10].

The endoscopic swallowing evaluation typically follows the FEES standard protocol, which may be adapted and expanded based on specific protocols tailored to particular tasks or clinical questions [2].

The final and intellectually most challenging part of the examination involves the multi-level interpretation of the diverse observations encountered during endoscopy and the deduction of clinical consequences. This review aims to provide guidance to meet this challenge by summarizing the necessary steps to systematically move from the salient findings to individualized diagnostic and therapeutic recommendations, thereby creating the framework of an integrated FEES report.

## Basic assessment

**Fig. 2:**
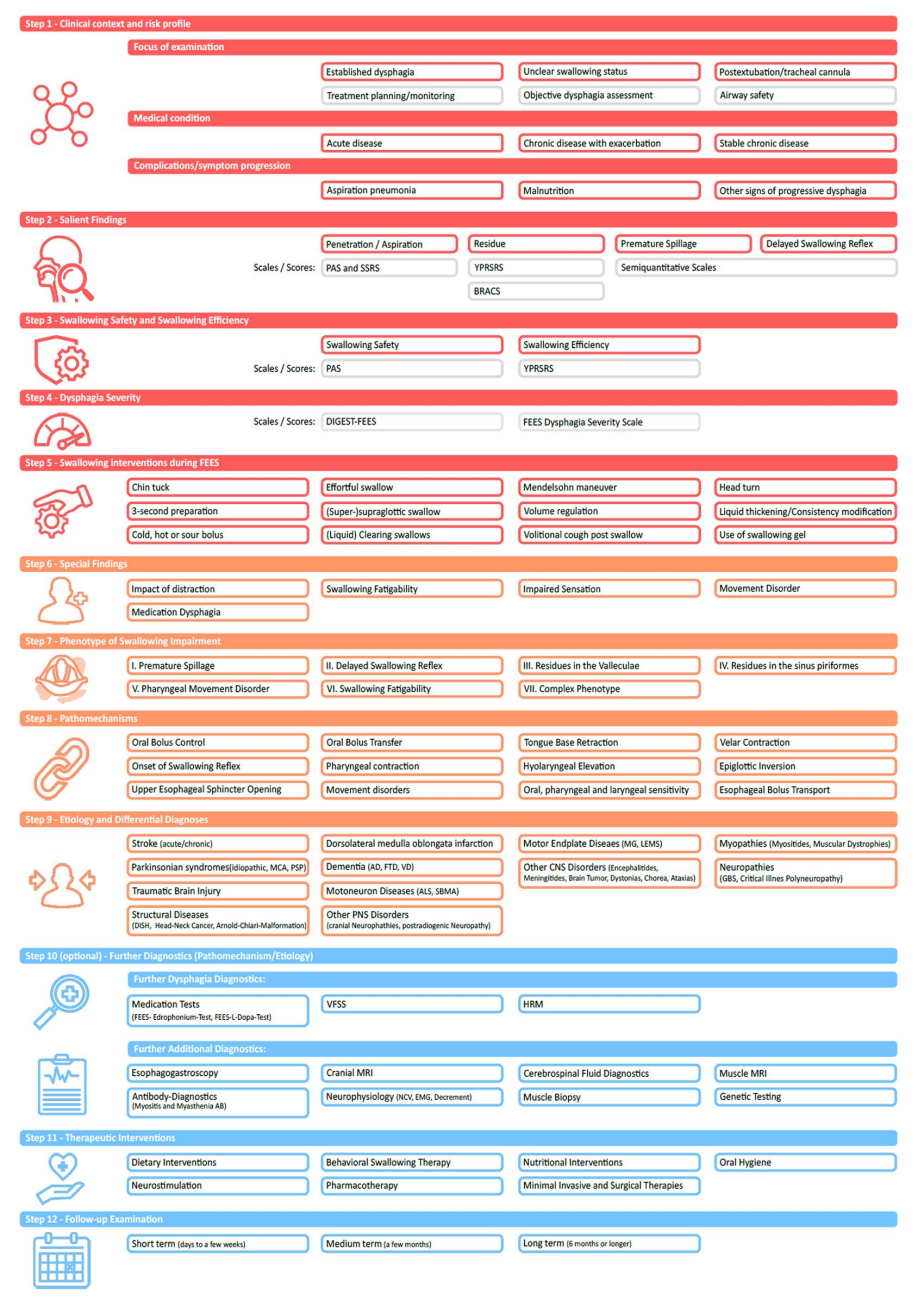
Framework of the integrated FEES report (Medicalgraphics.de; Cologne, Germany) (AD = Alzheimer‘s Dementia, BRACS = Boston Residue and Clearance Scale, DIGEST = Dynamic Grading of Swallowing Toxicity, DISH = Diffuse idiopathic skeletal hyperostosis, EMG = Electromyography, FTD = Frontotemporal Dementia, GBS = Gullain-Barré Syndrome, HRM = High Resolution Manometry, LEMS = Lambert-Eaton Myathenic Syndrome, MRI = Magnetic Resonance Imaging, MSA = Multiple System Atrophy, NCS = Nerve Conduction Study, PAS = Penetration Aspiration Scale, PNS = Peripheral Nervous System, PSP = Progressive Supranucelar Palsy, SBMA = Spinobulbar muscular atrophy, SSRS = Secretion Severity Rating Scale, VD = Vascular Dementia, VFSS = Videofluoroscopic Swallowing Study, YPRSRS = Yale Pharyngeal Residue Severity Scale)

As shown in Fig. 2, the first part of the integrated report focuses on the identification and grading of the key findings of the endoscopic swallowing assessment and is designed as a five-step approach.

### Step 1– Clinical context and risk profile

As prelude to FEES, the key elements of the clinical context at hand should be noted systematically. The first category assesses the focus of the examination. In the scenario of an already established dysphagia, FEES is usually carried out to plan further treatment or monitor the course of the disease. Patients with subjective swallowing complaints but no previous instrumental assessment are studied to objectively evaluate the swallowing function. Finally, in patients with a tracheal cannula or directly post-extubation the issue of airway safety is often in the focus of FEES. The second important differentiation refers to the time course of the illness. On the one hand the patient may be acutely ill, either due to the onset of a new disease, such as stroke, or due to the acute worsening of a chronic condition, such myasthenia gravis or Parkinson’s disease (i.e. myasthenic or akinetic crisis). On the other hand, FEES may take part in a stable medical condition, for example within a long-term scheduled outpatient counseling due to a slowly progressive swallowing impairment. The third dimension targets dysphagia-related complications and other clues from the patient’s history suggesting a relevant and short-term decline in swallowing function. To this end, it should be noted whether the patient has recently suffered episodes of aspiration pneumonia, experienced an unwanted weight-loss or reports other critical issues like change of diet and frequent coughing during mealtimes [10]. This principled distinction between a high-risk and a low-risk scenario is important since it impacts on the treatment strategies determined further down the line. In acute stroke, for example, aspiration observed during FEES substantially increases the short-term risk of aspiration pneumonia and therefore necessitates a very cautious feeding strategy often implementing temporary tube feeding [11, 12]. At the other end of the spectrum dysphagia with aspiration, which is detected in up to 20% of early-stage Parkinson’s disease [13] and 30% of patients with inflammatory myopathies [14], will be handled much less rigorously in these conditions, in particular if there is no indication of recent dysphagia-related complications.

### Step 2– The salient findings

According to Langmore the so called salient findings of FEES consist of premature spillage, delayed swallowing reflex, residue, penetration and aspiration [15, 16]. Salient findings should be described separately for all tested consistencies. In addition, a grading of severity should be provided. For rating of penetration and aspiration of bolus material the penetration-aspiration scale (PAS) may be used [17]. In order to semiquantitatively score penetration/aspiration of saliva the secretion severity rating scale (SSRS) may be applied [18]. Both the PAS and the SSRS have been shown to be valid and reliable [19–21]. Suggested scales for residue rating include the Yale Pharyngeal Residue Severity Rating Scale (YPRSRS) [22] and the Boston Residue And Clearance Scale (BRACS) [23]. Both scales have shown excellent reliability and validity, while up to now the YPRSRS seems to have achieved a more profound dissemination in the scientific community. Thus, the YPRSRS has been translated into a variety of different languages [24–26], has received independent testing for reliability and validity [27] and has been used in a high number of clinical trials [28–33]. Contrasting with this the BRACS, which is somewhat more complicated to score, has so far been only adopted in one study on dysphagia in Parkinson’s disease [34]. Semiquantitative assessment of premature spillage and delayed or absent swallowing reflex may be based on comparatively simple scores that have been shown reasonable interrater reliability. Thus, for characterizing premature spillage a 5-point scale was developed (0 = bolus is behind the tongue, 1 = bolus is at the base of the tongue or valleculae, 2 = the bolus moves to lateral channels or the tip of the epiglottis, 3 = the bolus is in the piriforms or touches the laryngeal rim, 4 = the bolus falls into the laryngeal vestibule) [35–37]. The timing of the swallowing reflex may be broken down into 0 = normal reflex latency, 1 = delay of swallowing reflex ≥ 3 s after the bolus has reached the valleculae, 2 = no swallowing reflex is elicited [37].

### Step 3– Swallowing safety and swallowing efficiency

Swallowing safety denotes the patient’s ability to protect the airway by a timely laryngeal vestibule closure [38]. Clinical correlates of impaired swallowing safety are cough, voice change, bolus aspiration with immediate respiratory distress and aspiration pneumonia. For assessing swallowing safety, the PAS score determined in step 1 is used. Swallowing efficiency refers to the ability to transport food and liquid through the oral cavity and the pharynx into the esophagus without post-swallow residues [38]. Bolus transfer through the alimentary tract is related to adequate pressure generation behind the bolus to allow sufficient propulsive force [39]. Patients with impaired swallowing efficiency may typically report the feeling of food getting stuck in the throat, the necessity to chew more carefully and to have repetitive clearing swallows and longer duration of mealtimes. Weight loss and malnutrition are the key complications of this impairment. For assessing swallowing efficiency, a residue scale, preferentially the YPRSRS, should be used. As conclusion of step 2 the main problem of the patient’s swallow within these coordinates should be noted. Thus, there may be (i) a single issue with either swallowing safety or efficiency, (ii) both domains may be impaired unrelated from each other, or (iii) swallowing safety maybe impaired because of critically reduced swallowing efficiency.

### Step 4– Dysphagia severity

As fourth step of the basic assessment a global rating of the observed swallowing impairment should be provided. This rating constitutes a summary observation, which helps, among others, to precisely and comprehensibly inform other health care professionals involved in the treatment of the patient of the severity of dysphagia and of potential changes over time. To date, the Dynamic Imaging Grade of Swallowing Toxicity (DIGEST), which has originally been conceived and refined for the rating of VFSS [40, 41], may be considered as an appropriate measures for this purpose. In the meantime, the DIGEST-FEES has been successfully validated by two independent groups [42, 43]. While this score has been developed for assessing dysphagia in head and neck cancer patients, it seems likely that it will be rapidly applied to other clinical contexts and patient populations. Thus, recently, the DIGEST-FEES has been successfully used to track the effectiveness of swallowing therapy in a cohort of chronic stroke victims [44] and has also been evaluated for potential use in patients with Parkinson’s disease [45]. In brief, the DIGEST-FEES is a five-point ordinal score that rates dysphagia at the patient level rather than on the bolus level and considers the two dimensions of swallowing safety and swallowing efficiency. The two-way interaction of swallowing safety and swallowing efficiency results in the DIGEST score, which classifies swallowing impairment into none (score 0), mild (score 1), moderate (score 2), severe (score 3) and life-threatening (score 4) [42].

As a somewhat less complex alternative the FEES dysphagia score may be used, which grades dysphagia on a 4-point scale into 0 = none, 1 = mild (premature spillage and/or residue but no penetration/aspiration), 2 = moderate (penetration/aspiration of one consistency) and 3 = severe (penetration/aspiration of two or more consistencies) [36]. This score has been adopted in different patient cohorts such as Parkinson’s disease [35], progressive supranuclear palsy [36], essential tremor [46], myasthenia gravis [47] and Guillain-Barré syndrome [48]. In addition, the multicenter prospective FEES-registry study showed that the FEES dysphagia score was highly correlated with the Functional Oral Intake Scale (FOIS) with scores of 0 and 1 being related to full oral intake levels with some degree of preparation and/or compensation, score of 2 being matched with FOIS level 4 indicating full oral nutrition with one consistency, and score of 3 requiring tube feeding with inconsistent oral intake [49]. In addition, there are scores for grading dysphagia in specific diagnostic groups, such as the fiberoptic endoscopic dysphagia severity scale (FEDSS) for acute stroke patients or the FEES-L-Dopa test for patients with Parkinsonian syndromes [36, 50].

### Step 5– Effect of swallowing interventions

A key advantage of FEES consists in the possibility of immediately testing swallowing maneuvers, head postures and bolus modifications to improve swallowing efficiency and swallowing safety [2, 51]. Table 1 provides a summary of key findings and related swallowing interventions applicable during FEES. Based on the individual impairment profile, suitable maneuvers should be applied during FEES and their effects noted in the FEES-report.

**Table 1:** Swallowing interventions to be tested during FEES depending on the key findings (based on [51, 88])

Finding	Maneuvers and strategies
Premature spillage + airway invasion	• 3-s preparation
	• Chin tuck
	• Volume regulation
	• Liquid thickening
	• (Super-)supraglottic swallow
Delayed swallowing reflex + airway invasion	• 3-s preparation
	• Chin tuck
	• Volume regulation
	• Liquid thickening
	• Cold or hot bolus
	• Sour bolus
	• (Super-)supraglottic swallow
Nasal regurgitation	• Volume regulation
	• Liquid thickening
Residues in the valleculae	• Consistency modifications
	• Effortful swallow
	• Mendelsohn maneuver
	• (Liquid) Clearing swallows
	• Chin tuck
Symmetrical residues in the sinus piriformes + airway invasion	• Consistency modifications
	• Mendelsohn maneuver
	• Head turn to either side
	• Effortful swallow
	• (Liquid) Clearing swallows
	• Volitional cough post swallow
Asymmetrical resides in the sinus piriformes + airway invasion	• Consistency modifications
	• Mendelsohn maneuver
	• Head turn to weaker side
	• Effortful swallow
	• (Liquid) Clearing swallows
	• Volitional cough post swallow
Medication dysphagia	• Depending on the concrete findings choose from the list above
	• Use of swallowing gel

## Advanced assessment

The second part of the integrated FEES-report covers advanced findings and interpretations of the endoscopic swallowing evaluation in four steps (Fig. 2).

### Step 6– Special findings

Special findings refer to distinctive features that are usually detected because the examiner has decided to carry out a specific FEES-protocol during or after running the FEES standard protocol. The additional protocols mentioned here, are easily implemented in the endoscopic swallowing evaluation and help answering specific questions that usually come up before or during the investigation [10].

#### Distraction

To assess the impact of distraction on swallowing performance the FEES-dual task protocol may be applied [52, 53]. In general, swallowing often occurs concurrently with mentally demanding activities, such as watching television or engaging in group conversations [54]. This is not reflected in the standard dysphagia examination, which mainly evaluates volitional swallowing in a more or less artificial examination context. As the simultaneous execution of two tasks (dual-task) often leads to delayed reaction times or deteriorated performances in one or both tasks—especially in patients with neurological disorders—exploring this aspect may be relevant when assessing swallowing in diverse patient groups and clinical settings. During the FEES-dual task paradigm the baseline FEES without interference is compared with a cognitive dual task on the one hand (memorizing a 6-digit number during the swallowing trials and recalling it thereafter) and a motor dual task (alternately using two click-devices with the right and left hand as often as possible during swallowing) on the other hand [53]. For comprehensively assessing swallowing function on a swallow-to-swallow basis the parameters “premature bolus spillage”, “penetration/aspiration” and “residue” are rated separately on established scales, each ranging from 0 (normal) to 4 (most severe). According to the protocol three boluses each of semisolid, liquid and solid food consistencies are evaluated and a cumulative score is generated by adding up the results of the single swallows, ranging from 0 (best) to 108 (worst) [52]. This dual task paradigm has been shown to have sufficient inter- and intra-rater reliability and helped to unmask central compensation of impaired swallowing in a cohort of patients with Parkinson’s disease [52].

#### Swallowing fatigability

Swallowing fatigability, which is characterized by a decline in swallowing function during the execution of repetitive swallows, is a key feature of myasthenic dysphagia and may, more rarely, be also encountered in patients with amyotrophic lateral sclerosis and other neuromuscular disorders. Muscle fatigue during swallowing typically develops over time and is most likely provoked by solid food, which requires a more effective pharyngeal squeeze than liquid or semisolid consistencies. Due to these facts swallowing fatigability may be missed during the execution of the standard FEES protocol, which typically includes only a limited number of swallows. To account for this the fatigable swallowing test (FST) has been developed [55]. For the FST the patient is given in succession up to 30 small pieces of white bread with a size of approximately 3 cm x 3 cm x 0.5 cm each. The number of successfully swallowed boluses until severe residues (> 50% of bolus size) occur, is used to quantify the severity of swallowing fatigability [55]. The FST has been attributed a reasonable sensitivity and specificity for differentiating myasthenic from non-myasthenic dysphagia [47].

#### Pharyngeal hypesthesia

Intact pharyngeal sensation is crucial for a physiological swallowing process, and conversely, pharyngeal hypesthesia is a key driver of dysphagia [56]. Pharyngeal hypesthesia can be caused both centrally, for example, by damage to somatosensory cortical areas, and peripherally, for example, by injury of the pharyngeal mucosa. Therefore, during FEES pharyngeal sensation should be carefully evaluated. The most common approach to indirectly assessing sensory function consists in determining the patient’s reaction to airway invasion and pharyngeal residue. Thus, the PAS differentiates between penetration and aspiration with and without subsequent ejection from the airway [17]. Additionally, the BRACS not only evaluates the extent and location of pharyngeal residues but also considers the lack of spontaneous clearing swallows as sign for impaired pharyngeal sensation [23]. A very straightforward method for investigating pharyngeal sensation is the so called “touch-technique”. This approach involves touching specific pharyngeal and laryngeal structures with the tip of the endoscope and score the patient’s reaction to this supraphysiological stimulus, which may consist in the laryngeal adductor reflex, swallowing and coughing [57, 58]. A more refined technique for sensory testing is the air pulse method (FEES with sensory testing, FEESST). Here, an air pulse is applied to the pharyngeal wall or laryngeal structures through an additional channel of the endoscope. The pressure of the air pulse can be varied and thus a threshold value is determined at which the laryngeal adductor reflex is triggered [59, 60]. The clinical relevance of FEESST has been discussed controversially, and conflicting results have been published [57, 61, 62]. The FEES-Laryngeal-Swallow-Response-Test (FEES-LSR-Test) also aims for a quantitative description of pharyngeal hypesthesia but, in comparison to FEESST, makes use of a physiological stimulus. For this test a small tube is positioned transnasally in the upper third of the oropharynx with contact to the lateral pharyngeal wall. Increasing volumes of dyed water are injected through the tube, and the latency of swallowing response is determined endoscopically [63]. This test was validated in healthy subjects and clearly distinguished between the physiological state and experimentally induced pharyngeal anesthesia, in addition inter- and intra-rater reliability were excellent [63]. In further studies pharyngeal hypesthesia as documented by the FEES-LSR-Test was correlated with dysphagia severity both in stroke victims [64] and in community-dwelling older adults [65].

#### Movement disorders

Dysphagia is a clinical hallmark of many movement disorders such as Parkinson’s disease (PD) and atypical Parkinsonian syndromes [66, 67]. In addition to obvious swallowing difficulties, there are also more subtle indicators of abnormal movement patterns that may provide valuable clues in the diagnostic workup of patients and should be specifically addressed during FEES. *Oropharyngeal bradykinesia* is a frequent symptom in PD patients and may lead to decreased oral bolus control, prolonged transit times during swallowing, premature bolus spillage, drooling of saliva, delayed laryngeal vestibule closure, or aspiration [68]. Endoscopically, bradykinesia may manifest by increased duration of the white-out. While the average length of white-out in older subjects was reported to be 675 ms, PD patients had a white-out duration of 984 ms, which decreased with increasing intestinally supplied dopaminergic dosage to approximately 700 ms [69, 70]. Oropharyngeal freezing (OPF) is another movement pathology occurring during the swallow, which may be present in around one third of PD patients [71]. OPF shares common characteristics of the well-known freezing of gait, which refers to a sudden und temporarily interruption of motion during gait. As described in detail elsewhere [71], three subtypes of OPF have been described. Oropharyngeal festination means that the tongue base performs quick pumping movements that result in inadequate bolus propulsion. Oropharyngeal trembling refers to an ineffective shaking of the tongue and the pharyngeal constrictors. Finally, oropharyngeal akinesia characterizes a complete interruption of the swallowing process. In this condition, the bolus has reached the trigger zones of the swallowing reflex in the hypopharynx, yet, momentarily, no swallow response is elicited. In contrast to these findings that are typically observed during the swallow, specific laryngeal movement abnormalities during a set of dedicated positioning tasks of laryngeal function are considered characteristic of multiple system atrophy (MSA) [72, 73]. Thus, more than 90% of MSA patients showed an irregular arytenoid cartilage movement (iACM), which is an irregular jitter and flutter of the arytenoid region occurring during breathing and phonation, while iACM were not seen in any of the studied PD patients [73]. Hence, this MSA-protocol should be added to the standard physiological assessment of FEES, if a movement disorder is suspected in the respective patient.

#### Swallowing medication

Many patients with dysphagia encounter challenges when taking oral medication, particularly swallowing tablets and capsules. Apart from aspiration and associated complications, patients may discontinue their medication or make unsuitable modifications due to these difficulties (e.g., crushing, breaking, and opening of tablets and capsules). The latter may lead to numerous problems, such as decreased accuracy of dose, increased toxicity, reduced stability, and alteration of pharmacokinetics [74]. As per recent guidelines, it is recommended that swallowing of tablets be routinely assessed as part of the swallowing evaluation, particularly for patients with dysphagia who need to take oral medication [10, 12], and the most suitable form of oral administration should be identified. For the classification of medication dysphagia, a dedicated score that specifically evaluates the efficiency and safety of swallowing pills and capsules may be used [75]. This 5-point ordinal score has been attributed a high interrater reliability both for the subscale of swallowing efficiency (κ = 0.89) and swallowing safety (κ = 0.86). With regards to validity this score was significantly linked to the occurrence of motor complications in a cohort of 66 PD patients [75].

### Step 7– Phenotypes of swallowing impairment

Based on the salient findings and taking into consideration the results of the advanced assessment of step 6, the phenotype of swallowing impairment should be determined. A phenotypic classification of neurogenic dysphagia that may be used for this purpose was developed by means of a systematic literature review, fine-tuned by an additional analysis of 200 selected FEES videos and validated using more than 1000 additional FEES videos from a variety of different neurological diseases [37]. As a result of this analytical process the following seven phenotypes were derived: (1) premature bolus spillage, (2) delayed swallowing reflex, (3) predominance of residue in the valleculae, (4) predominance of residues in the piriform sinus, (5) pharyngeal movement disorder, (6) fatigable swallowing weakness, and (7) complex phenotye. Some of these phenotypes, such as premature spillage, residue in the valleculae, or the complex pathology occurred in many neurological diseases and can therefore be considered as transdiagnostic patterns in neurogenic dysphagia. Other phenotypes such as residue with predominance in the piriform sinus, impaired swallowing reflex, pharyngolaryngeal movement disorders, and fatigable swallowing weakness may serve as indicators for specific diseases [37]. Based on these findings, Table 2 matches the different phenotypes to diseases that affect the central nervous system or peripheral components of the swallowing network. The interrater agreement of this phenotypic classification of FEES findings was strong (κ = 0.84) [37].

**Table 2:** Typical FEES-phenotypes in different neurological disorders (modified from [37])

Main findings	Neurological diseases	
	Peripheral	Central
I) Premature spillage	Early-stage ALS	Acute stroke, early-stage ALS, early-stage PSP, frontotemporal dementia, HSP
II) Delayed swallowing reflex		Acute stroke
III) Impaired pharyngeal bolus clearance (residue in valleculae >>> residue in piriform sinus)	Bulbospinal muscular atrophy, myotonic dystrophy type II, CIP/CIM, early-stage ALS	Early ALS, early-stage PD
IV) Impaired pharyngeal bolus clearance (residue in piriform sinus >>> residue in valleculae)	Inclusion body myositis	Dorsolateral medulla oblongata infarction
V) Extrapyramidal motor impairment (one out of I-IV) plus movement disorder	–	Neuroleptic-induced dysphagia, PD, MSA, Huntington’s disease, mesencephal or basal ganglia stroke
VI) Fatigable oropharyngeal dysphagia (one out of I-IV plus swallowing fatigability)	Myasthenia gravis	ALS
VII) Complex pathology (combination of I-IV, at least 2 equivalent patterns)	Severe myasthenia gravis, end-stage ALS, GBS, myotonic dystrophy type I	End-stage ALS, advanced stages of PD and PSP

ALS = Amyotrophic Lateral Sclerosis, CIP/CIM = Critical Illness Polyneuropathy/Myopathy, GBS = Guillain-Barré syndrome, HSP = Hereditary Spastic Paraparesis, MSA = Multiple System Atrophy, PD Parkinson’s Disease, PSP = Progressive Supranuclear Palsy

### Step 8– Pathomechanism

In this step, FEES findings characterizing the key aspects of the patient’s swallowing impairment need to be scrutinized with regards to the underlying pathophysiology. This point will be particularly important for designing an appropriate treatment plan later (see step 11). First, as summarized in Table 3, findings from the physiological examination should be considered. These non-swallow observations should then be matched with results of the swallow studies. Here, as already stressed by Susan Langmore in her pertinent book and updated in two recent reviews by the same author and Schindler and colleagues respectively [2, 15, 51], most relevant clues are provided by analyzing the distribution of pharyngeal residues. For example, residues at the tongue base or in the valleculae are most often caused by poor tongue base retraction. Residues in the piriform sinus suggest impaired opening of the upper esophageal sphincter, weak pharyngeal contraction and reduced hyolaryngeal elevation. Next, if present, the timing of airway invasion should be looked at. Finally, there are several additional observations during the swallow assessment that help clarifying the individual patient’s pathophysiology. For example, the frequently encountered phenomenon of preswallow pharyngeal spillage may be due to both, an impaired oral bolus control and a delayed swallowing reflex. Apart from that, however, also the less common and more difficult to classify conditions of akinesia of swallowing [71] and palatal myoclonus can be relevant here [76]. Recently, the white-out and its changes from normal have been studied more closely. Thus, as already mentioned above, a prolonged white-out may be due to pharyngeal bradykinesia, a hallmark of PD-related dysphagia [69], and, presumably, may also be seen in pharyngeal dystonia. A weak white-out in turn, which can be classified with a simple ordinal scale [14], is related to a weakened pharyngeal contraction and, possibly, impaired retraction of the tongue-base. The so-called postswallow-stage, which immediately follows the white-out, may also provide clues for deciphering the underlying pathophysiology. According to a recent study analyzing a set of simultaneous VFSS-FEES examinations, incomplete epiglottic inversion was related to a combined effect of reduced tongue-base retraction, impaired hyo-laryngeal elevation and weakened pharyngeal contraction [77].

**Table 3:** Clinical observations during FEES and related pathophysiology (based on reviews [2, 15, 51], new references added where appropriate)

Clinical observations	Pathophysiology
**Non-swallow observations**	
• Specific impairments during motor and sensory tasks	Velopharyngeal contraction↓, dystonic pharyngeal contraction [89], pharyngeal contraction↓, tongue base retraction↓, laryngeal closure↓, laryngeal and pharyngeal hypesthesia
**Location of residue**	
• Nasopharynx	Velopharyngeal contraction↓
• Base of the tongue	Tongue base retraction↓
• Valleculae	Tongue base retraction↓
	Hyolaryngeal elevation↓
	Pharyngeal bradykinesia [71]
• Pyriform sinus	Pharyngeal contraction↓
	Hyolaryngeal elevation↓
	Opening of the UES↓
• Postcricoid region	Opening of the UES↓
	Bolus flow through upper esophagus↓
• Pharyngeal wall	Pharyngeal contraction↓
• Laryngeal epiglottis	Incomplete glottic closure
**Timing of airway invasion**	
• Preswallow penetration/aspiration	Preswallow pharyngeal spillage (see below)
• Intraswallow penetration/aspiration	Delayed or incomplete glottic closure
• Postswallow penetration/aspiration	Residues/Regurgitation with overflow in the laryngeal vestibule (see below)
**Further swallow observations**	
• Prolonged oral preparation/transport	Oral bolus control↓
	Oral bolus transfer↓
• Multiple Swallows per bolus (piecemeal deglutition)	Festination of swallowing [71]
• Preswallow pharyngeal spillage	Oral bolus control↓
	Swallowing reflex↓
	Akinesia of swallowing [71]
	Palatal myoclonus [76]
• Prolonged White-out	Pharyngeal dystonia
	Pharyngeal bradykinesia [69]
• Weak/missing white-out	Tongue base retraction↓
	Pharyngeal contraction↓ [14]
• Incomplete epiglottic inversion	Hyolaryngeal elevation↓
	Tongue base retraction↓
	Pharyngeal contraction↓ [77]
• Prolonged laryngeal reconfiguration	Pharyngeal bradykinesia [69]
	Pharyngeal dystonia
• Early esophago-pharyngeal reflux (rising-tide sign)	Bolus flow through upper esophagus↓
	Opening of the UES↓
• Delayed esophago-pharyngeal Regurgitation	Bolus flow through middle/lower esophagus↓
	Gastro-esophageal reflux
• Insufficient reaction to residue	Pharyngeal hypesthesia
• Insufficient reaction to penetration/aspiration	Laryngeal/tracheal hypesthesia

UES = upper esophageal sphincter

### Step 9– Etiology and differential diagnoses

This step is of particular relevance if the etiology of dysphagia in the patient at hand has not been determined yet. In this case the phenotypic classification (step 7) and pathophyisiological considerations (step 8) may be used to put forward a comprehensive list of possible differential diagnoses [10]. However, even in patients with a known diagnosis typically associated with swallowing impairments, it should be carefully checked, whether the observed dysphagia phenotype is compatible with this diagnosis.

## Recommendations

This concluding section of the integrated FEES-report aims to provide precise recommendations for subsequent diagnostic and therapeutic procedures as well as for the timing for a follow-up examination (Fig. 2).

### Step 10 - Further diagnostics to elucidate the pathophysiology and/or etiology of dysphagia

This step is optional and only applies if either the etiology or the pathophysiology of dysphagia after FEES is still unclear and needs to be clarified.

#### Additional dysphagia diagnostics

These include FEES-based medication tests, VFSS and high-resolution manometry (HRM).

FEES-based medication tests evaluate whether a swallowing impairment improves after application of a specific pharmaceutical agent. The FEES-Edrophonium test, which incorporates FEES with simultaneous intravenous application of Edrophonium during a standardized swallowing task, has been designed to uncover myasthenic dysphagia [78]. This test has been shown to be highly reliable [79] and, both, sufficiently sensitive and specific [47]. Due to these robust clinical properties the FEES-Edrophonium test is key part of an algorithm for the diagnostic workup of neurogenic dysphagia of undetermined etiology [80]. The FEES-L-Dopa test evaluates whether dysphagia in patients with parkinsonian syndromes improves after application of a sufficiently high dose of fast-dissolving L-Dopa. For semiquantitative rating of dysphagia severity the same protocol as mentioned in the section dealing with the dual-task paradigm is used. The FEES-L-Dopa test has been shown to be applicable without complications in the clinical routine and was attributed excellent inter- and intra-rater reliability [35, 36].

VFSS is a contrast based, radiological examination of the entire swallowing act including oral, pharyngeal, and esophageal stages. Apart from qualitative parameters VFSS also offers a variety of quantitative measures, such as the oral onset time, the oral transit time, the pharyngeal transit time, the anterior-superior movement of the hyoid, the duration of the velopharyngeal closure and the duration and width of the opening of the upper esophagus sphincter. Based on VFSS it is possible to detect and to comprehensively describe complex pathomechanisms of swallowing disorders affecting laryngo-pharyngeal and -esophageal interactions [10]. Therefore, according to a recent guideline, VFSS should be considered in particular, if based on FEES a complex pathophysiology of the pharyngeal phase, an opening disorder of the upper esophageal sphincter or an impairment of the esophageal phase is suspected [10].

HRM allows the endoluminal pressure conditions in the pharynx and esophagus to be measured during the swallowing act. The method is particularly suitable to prove relaxation disorders of the upper esophageal sphincter, for example after dorsolateral medulla oblongata infarction or in the course of inflammatory myopathies, and motility disorders of the tubular or lower esophagus, such as achalasia and diffuse esophagospasm. For esophageal HRM normal values have been established for the following parameters: resting pressure, duration of upper and lower esophageal sphincter opening as well as peristalsis, pressure and amplitudes of the tubular esophagus. Although there is still a lack of normative data, pharyngeal HRM has been used in different neurological diseases, such as stroke, PD and myopathies to decipher patterns of swallowing impairment [10].

#### Additional non-swallow diagnostics

If, based on the FEES findings, one specific disorder or a set of differential diagnoses is considered, further (non-swallow) diagnostic steps may be planned to determine the etiology of dysphagia. A summary line-up of diseases and related diagnostics is provided in Table 4. For example, in case of a suspected motoneuron disorder with bulbar onset dedicated electromyography should be scheduled. If oculopharyngeal muscular dystrophy needs to be considered, e.g. due to a positive family history, genetic testing is warranted. In cases where an acute inflammatory polyneuropathy comes into account, lumbar puncture and neurography may be the next diagnostic steps at hand. Finally, as outlined elsewhere in detail, the diagnostic-workup of suspected inflammatory myopathies includes antibody-testing, neurophysiological studies, magnetic resonance imaging of the muscles and a muscle biopsy, among others [80].

**Table 4:** Neurological diseases featuring dysphagia as possible main symptom, and important additional diagnostics [90]

Neurological disease	Additional diagnostics
Brainstem infarction	Brain MRI incl. DWI sequence
Listeria brainstem encephalitis	Brain MRI, lumbar puncture
Paraneoplastic brainstem encephalitis	Brain MRI, anti-neuronal antibodies (Hu, Ta, Ma, Ri), CV2/ anti-CRMP5, anti-amphiphysin, ANNA-3)
Brainstem tumor	Brain MRI, lumbar puncture with cytological examination, brain biopsy
Meningeosis neoplastica	Brain MRI, lumbar puncture with cytological examination
Basal meningitis	Lumbar puncture
Special forms of Guillain– Barré syndrome	Lumbar puncture, ganglioside antibodies (GD1a, GM1b, GW1b, GT1a)
Post-polio syndrome	EMG
Pseudobulbar paralysis	Brain MRI
Amyotrophic lateral sclerosis	NCS, EMG
Presbyphagia	Exclusion diagnostics
Skull base tumors	Brain MRI
Arnold–Chiari malformation, type I	Brain MRI
Neuroleptic-induced dysphagia	Medication history, exclusion diagnostics of other extrapyramidal motor diseases (if necessary)
Polymyositis	Serum creatinine kinase, EMG, muscle MRI, specific antibodies, muscle biopsy
Inclusion body myositis	Serum creatinine kinase, EMG, muscle MRI, specific antiboidies, muscle biopsy
Myasthenia gravis	EMG with low-frequency repetitive stimulation (3 Hz), Edrophonium-test, specific antibodies, Thorax CT
Lambert–Eaton myasthenic syndrome	EMG with high-frequency repetitive stimulation (10–50 Hz), antibodies against voltage-gated calcium channels
Botulism	History, toxin detection in body liquids
Tetanus	History, EMG, toxin detection in body liquids
Oculopharyngeal muscular dystrophy	Family history, genetics (PABP2-Gen)
Myotonic dystrophy, type I	Family history, EMG (myotonic discharges), genetics (CTG expansion in myotonin-protein kinase gene)
Post surgery or other interventions	History
Functional dysphagia	Exclusion diagnostics, psychiatric and psychosomatic evaluation

AChR = Acetylcholin Receptor, EMG = Electromyography, MRI = Magnetic Resonance Imaging, NCS = Nerve Conduction Study, MuSK = Muscle-specific Tyrosine Kinase

### Step 11– Therapeutic interventions

As summarized in a recent guideline, a variety of different therapeutic methods is now available for the treatment of neurogenic dysphagia [10]. Principally, the indication for a specific treatment is determined by both, the pathophysiology and the etiology of dysphagia, which is why the preceding diagnostic work-up is of key importance.

Therapeutic strategies include on the one hand protective and compensatory interventions, which aim at reducing the risk of dysphagia-related complications without targeting swallowing function itself. This category comprises of dietary interventions, in particular prescription of texture-modified food and thickened liquids, oral hygiene, i.e. consistent oral and dental cleaning, nutritional interventions, such as use of high-caloric oral supplementation or initiation of tube-feeding, and compensatory behavioral swallowing interventions, like the chin-down posture or the head-turn [10]. On the other hand, mainly driven by more recent clinical research, there is an expanding armamentarium of restorative treatment options available, which aim at improving swallowing physiology itself. Apart from restorative behavioral swallowing interventions, for example the Shaker exercise, Chin-tuck-against-resistance or expiratory muscle strength training, different neurostimulation modalities such as transcranial direct current stimulation, pharyngeal electrical stimulation or neuromuscular electrical stimulation need to be mentioned here. In addition, there is a growing body of evidence supporting the use of different pharmacological agents like capsaicinoids, and, finally, in selected patients minimally invasive or surgical procedures come into consideration, in particular balloon-dilatation or myotomy in case of cricopharyngeal dysfunction [10].

### Step 12– Follow-up examination

Scheduling the follow-up examination constitutes the final step of the integrated FEES-report. While in some patients no further FEES is necessary, for example at the end of a successful rehabilitation or in cases where the suspicion of dysphagia was not confirmed, many patients will have to be re-evaluated further down the line. The interval to the next swallowing assessment is determined by a variety of factors, such as the nature and the expected course of the disease, the treatment performed and the occurrence of complications. Thus, for example, in acute stroke, dysphagia is often severe at the onset of the disease, necessitating dietary adjustments or even tube feeding, but may improve rapidly within the first two weeks [11]. In such a clinical scenario, a follow-up assessment should be scheduled within the first week, which is also recommended by guidelines [81]. In stroke victims with a more complicated course, serial FEES may be required early on to adequately manage the swallowing impairment and its complications [82]. At the other end of the spectrum, there are patients with neurogenic dysphagia due to slowly progressive neurodegenerative disorders like PD. In a clinically stable situation regular follow-up examinations may take place in longer intervals, for example once a year or once every two years. However, the situation obviously changes if complications like an akinetic crisis occur, or the patient presents with new onset of motor-fluctuations indicating disease progression also typically affecting the swallowing function [68]. Finally, a medium term follow-up should be considered, among others, in chronic but more rapid-progressive neurodegenerative disorders, such ALS [83, 84], diseases with fluctuating clinical features, in particular Myasthenia gravis [47], and after specific interventions with unclear long-term effects, for example dilatation of the cricopharyngeal muscle in patients with myositis [85].

## Conclusions

Today, due to the complexity of the condition, patients with neurogenic dysphagia are supposed to be managed by multiprofessional teams [10]. While potential patient-centered benefits are obvious, any team-approach increases the need for targeted, comprehensive and comprehensible communication [86, 87]. The framework for creating an integrated FEES-report presented in this article was designed to foster, clarify and improve interaction in the multiprofessional team. The structured approach systematically moves from salient findings to more advanced domains such as dysphagia severity, phenotypes of swallowing impairment and pathomechanisms. That way, whenever available, validated scales and scores were used to increase the diagnostic yield of the report. Finally, FEES-findings are put into the perspective of the clinical situation at hand, thereby integrating the patient’s clinical situation into the swallowing-specific observations. Here, the potential etiology of dysphagia and conceivable differential diagnoses are considered, further diagnostic steps are proposed, treatment options are evaluated, and a re-assessment is scheduled.

Already by its design this framework is open to continuous evolution. Any new items, for example further FEES-protocols and pathophysiological observations, improved disease-related knowledge and novel treatment options will easily be incorporated. In addition, it is conceivable that this approach may also be customized to report on FEES in structural dysphagia.

## Data Availability

Not applicable.

## Abbreviations

AChR: Acetylcholin Receptor
AD: Alzheimer‘s Dementia
ALS: Amyotrophic Lateral Sclerosis
BRACS: Boston residue and clearance scale
CIP/CIM: Critical Illness Polyneuropathy/Myopathy
DISH: Diffuse idiopathic skeletal hyperostosis
DIGEST: Dynamic Imaging Grade of Swallowing Toxicity
EMG: Electromyography
ENG: Electroneurography
FEES: Flexible Endoscopic Evaluation of Swallowing
FEES-LSR-Test: FEES-Laryngeal-Swallow-Response-Test
FEESST: FEES with Sensory Testing
FOIS: Functional Oral Intake Scale
FST: Fatigable Swallowing Test
FTD: Frontotemporal Dementia
GBS: Guillain-Barré syndrome
HRM: High Resolution Manometry
HSP: Hereditary Spastic Paraparesis
iACM: irregular Arytenoid Cartilage Movement
LEMS: Lambert-Eaton Myathenic Syndrome
MRI: Magnetic Resonance Imaging
MSA: Multiple System Atrophy
MuSK: Muscle-specific Tyrosine Kinase
NCS: Nerve Conduction Study
OPF: Oropharyngeal freezing
PAS: Penetration-Aspiration Scale
PD: Parkinson’s Disease
PNS: Peripheral Nervous System
PSP: Progressive Supranuclear Palsy
SBMA: Spinobulbar muscular atrophy
SLP: Speech and Language Pathologist
SSRS: Secretion Severity Rating Scale
UES: Upper Esophageal Sphincter
VD: Vascular Dementia
VFSS: Videofluoroscopic Swallowing Study
YPRSRS: Yale pharyngeal residue severity rating scale
